# Penta­aqua­[2-(5-carboxyl­ato-2-oxido-1-pyridinio)acetato]zinc(II) monohydrate

**DOI:** 10.1107/S1600536810017538

**Published:** 2010-06-09

**Authors:** Jing Chen, Yun-Long Feng

**Affiliations:** a Zhejiang Key Laboratory for Reactive Chemistry on Solid Surfaces, Institute of Physical Chemistry, Zhejiang Normal University, Jinhua, Zhejiang 321004, People’s Republic of China

## Abstract

In the title compound, [Zn(C_8_H_5_NO_5_)(H_2_O)_5_]·H_2_O, the Zn^II^ atom is coordinated by one O atom from the 2-(5-carboxyl­ato-2-oxidopyridinium-1-yl)acetate ligand and by five water mol­ecules, forming a distorted octa­hedral geometry. Coordinated and uncoordinated water mol­ecules form O—H⋯O hydrogen bonds, leading to a three-dimensional framework.

## Related literature

For related structures, see: Jiang *et al.* (2009 ▶); Szafran *et al.* (2006 ▶); Yang *et al.* (2010 ▶); Zhang *et al.* (2003 ▶); He & Feng (2007 ▶).
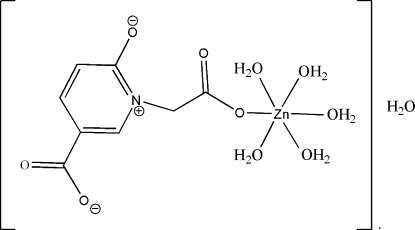


## Experimental

### 

#### Crystal data

[Zn(C_8_H_5_NO_5_)(H_2_O)_5_]·H_2_O
*M* *_r_* = 368.60
Monoclinic, 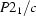

*a* = 10.9584 (4) Å
*b* = 7.5548 (4) Å
*c* = 16.6510 (7) Å
β = 103.498 (3)°
*V* = 1340.43 (10) Å^3^
*Z* = 4
Mo *K*α radiation
μ = 1.89 mm^−1^
*T* = 293 K
0.36 × 0.09 × 0.05 mm

#### Data collection

Bruker APEXII area-detector diffractometer
Absorption correction: multi-scan (*SADABS*; Sheldrick, 1996 ▶) *T*_min_ = 0.824, *T*_max_ = 0.918
19343 measured reflections
3086 independent reflections
2233 reflections with *I* > 2σ(*I*)
*R*_int_ = 0.100

#### Refinement

*R*[*F* ^2^ > 2σ(*F* ^2^)] = 0.036
*wR*(*F* ^2^) = 0.084
*S* = 1.00
3086 reflections
226 parameters
18 restraints
H atoms treated by a mixture of independent and constrained refinement
Δρ_max_ = 0.48 e Å^−3^
Δρ_min_ = −0.80 e Å^−3^

### 

Data collection: *APEX2* (Bruker, 2006 ▶); cell refinement: *SAINT* (Bruker, 2006 ▶); data reduction: *SAINT*; program(s) used to solve structure: *SHELXS97* (Sheldrick, 2008 ▶); program(s) used to refine structure: *SHELXL97* (Sheldrick, 2008 ▶); molecular graphics: *SHELXTL* (Sheldrick, 2008 ▶); software used to prepare material for publication: *SHELXTL*.

## Supplementary Material

Crystal structure: contains datablocks I, global. DOI: 10.1107/S1600536810017538/is2541sup1.cif

Structure factors: contains datablocks I. DOI: 10.1107/S1600536810017538/is2541Isup2.hkl

Additional supplementary materials:  crystallographic information; 3D view; checkCIF report

## Figures and Tables

**Table 1:** Hydrogen-bond geometry (Å, °)

*D*—H⋯*A*	*D*—H	H⋯*A*	*D*⋯*A*	*D*—H⋯*A*
O1*W*—H1*WA*⋯O2^i^	0.83 (2)	2.25 (2)	2.972 (3)	145 (3)
O1*W*—H1*WB*⋯O2*W*^ii^	0.81 (2)	2.58 (3)	3.118 (3)	125 (3)
O2*W*—H2*WA*⋯O4^iii^	0.83 (2)	1.89 (2)	2.716 (2)	169 (3)
O2*W*—H2*WB*⋯O2^i^	0.80 (2)	1.96 (2)	2.742 (2)	163 (2)
O3*W*—H3*WA*⋯O2^iv^	0.81 (2)	1.89 (2)	2.701 (2)	173 (2)
O3*W*—H3*WB*⋯O4^v^	0.82 (2)	2.04 (2)	2.849 (2)	170 (3)
O4*W*—H4*WA*⋯O5^vi^	0.82 (2)	2.02 (2)	2.810 (2)	160 (3)
O5*W*—H5*WA*⋯O1^iii^	0.82 (2)	1.90 (2)	2.705 (2)	166 (3)
O5*W*—H5*WB*⋯O1^iv^	0.81 (2)	1.95 (2)	2.742 (2)	164 (3)
O6*W*—H6*WB*⋯O3^v^	0.82 (2)	1.89 (2)	2.697 (3)	171 (3)

Symmetry codes: (i) 
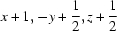
; (ii) 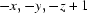; (iii) 
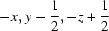
; (iv) 
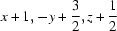
; (v) 
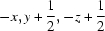
; (vi) 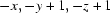.

**Fig. 1.**
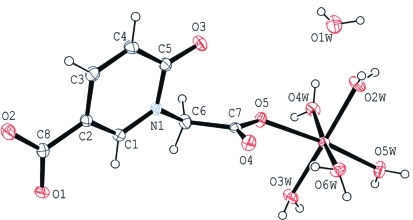
The molecular structure of the title compound, with 30% probability displacement ellipsoids.

**Fig. 2.**
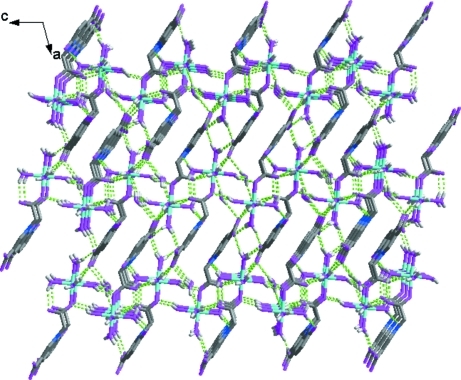
Three-dimensional framework with hydrogen bonding interactions.

## supplementary crystallographic information

### Comment

The pyridinium carboxylate ligands, containing both of carboxylate and quaternary ammonium groups, have been extensively employed to design and construct novel complexes due to its versatile coordination behavior to metal ions (Zhang *et al.*, 2003; Szafran *et al.*, 2006; Yang *et al.*, 2010). Herein, we report the synthesis and crystal structure of a new complex, [ZnL(H_2_O)_5_].H_2_O (LH_2_ = 5-carboxy-1-carboxymethyl-2-oxidopyridinium; He & Feng, 2007).

As shown in Fig. 1, the metal center Zn^II^ atom is six-coordinated by one O atom from one L^2-^ ligand [Zn—O 2.1039 (16) Å] and five water molecules [Zn—O 2.0554 (17)–2.0988 (19) Å], to form a distorted octahdral geometry. Notably, only one O atom from the flexible carboxylic groups of L^2-^ ligand coordinates to the Zn^II^ ion. As shown in Fig. 2, the complexes connected with each other by the O—H···O hydrogen bonds generate a three-dimensional structure.

### Experimental

All the starting materials and solvents were obtained commercially and were used without further purification. A mixture of *N*-carboxymethyl-2-oxo-pyridine-5-carboxylic acid (0.1972 g, 1 mmol), ZnNO_3_ (0.1901 g, 1 mmol), and purified water (15 ml) was sealed in a 25 ml stainless steel reactor and kept at 393 K for 3 d. Then, the reactor was cooled to room temperature at a speed of 5 K/h. A large quantity of colorless single crystals were filtered out of the mixture with the yield of 85%.

### Refinement

The C-bound H atoms were positioned geometrically and included in the refinement using a riding model, with C—H = 0.93 or 0.97 Å, and with *U*_iso_(H) = 1.2*U*_eq_(C). The O-bound H atoms was located in a difference Fourier map and refined, with the distance restraint of O—H = 0.82 (2) Å, and with *U*_iso_(H) = 1.2*U*_eq_(O).

### Crystal data

**Table:**

[Zn(C_8_H_5_NO_5_)(H_2_O)_5_]·H_2_O	*F*(000) = 760
*M**_r_* = 368.60	*D*_x_ = 1.826 Mg m^−^^3^
Monoclinic, *P*2_1_/*c*	Mo *K*α radiation, λ = 0.71073 Å
Hall symbol: -P 2ybc	Cell parameters from 6642 reflections
*a* = 10.9584 (4) Å	θ = 1.9–27.6°
*b* = 7.5548 (4) Å	µ = 1.89 mm^−^^1^
*c* = 16.6510 (7) Å	*T* = 293 K
β = 103.498 (3)°	Prism, colourless
*V* = 1340.43 (10) Å^3^	0.36 × 0.09 × 0.05 mm
*Z* = 4	

### Data collection

**Table:**

Bruker APEXII area-detector diffractometer	3086 independent reflections
Radiation source: fine-focus sealed tube	2233 reflections with *I* > 2σ(*I*)
graphite	*R*_int_ = 0.100
ω scans	θ_max_ = 27.6°, θ_min_ = 1.9°
Absorption correction: multi-scan (*SADABS*; Sheldrick, 1996)	*h* = −14→14
*T*_min_ = 0.824, *T*_max_ = 0.918	*k* = −9→8
19343 measured reflections	*l* = −21→19

### Refinement

**Table:**

Refinement on *F*^2^	Primary atom site location: structure-invariant direct methods
Least-squares matrix: full	Secondary atom site location: difference Fourier map
*R*[*F*^2^ > 2σ(*F*^2^)] = 0.036	Hydrogen site location: inferred from neighbouring sites
*wR*(*F*^2^) = 0.084	H atoms treated by a mixture of independent and constrained refinement
*S* = 1.00	*w* = 1/[σ^2^(*F*_o_^2^) + (0.0382*P*)^2^] where *P* = (*F*_o_^2^ + 2*F*_c_^2^)/3
3086 reflections	(Δ/σ)_max_ < 0.001
226 parameters	Δρ_max_ = 0.48 e Å^−^^3^
18 restraints	Δρ_min_ = −0.79 e Å^−^^3^

### Special details

**Table:**

Geometry. All esds (except the esd in the dihedral angle between two l.s. planes) are estimated using the full covariance matrix. The cell esds are taken into account individually in the estimation of esds in distances, angles and torsion angles; correlations between esds in cell parameters are only used when they are defined by crystal symmetry. An approximate (isotropic) treatment of cell esds is used for estimating esds involving l.s. planes.
Refinement. Refinement of *F*^2^ against ALL reflections. The weighted *R*-factor wR and goodness of fit *S* are based on *F*^2^, conventional *R*-factors *R* are based on *F*, with *F* set to zero for negative *F*^2^. The threshold expression of *F*^2^ > σ(*F*^2^) is used only for calculating *R*-factors(gt) etc. and is not relevant to the choice of reflections for refinement. *R*-factors based on *F*^2^ are statistically about twice as large as those based on *F*, and *R*- factors based on ALL data will be even larger.

### Fractional atomic coordinates and isotropic or equivalent isotropic displacement parameters (Å^2^)

**Table:**

	*x*	*y*	*z*	*U*_iso_*/*U*_eq_	
Zn1	0.14951 (2)	0.39964 (4)	0.404996 (17)	0.02275 (11)	
O1	−0.56142 (14)	0.8182 (2)	0.07024 (11)	0.0314 (4)	
O1W	0.0969 (2)	0.0417 (3)	0.59084 (15)	0.0564 (6)	
H1WA	0.1593 (19)	−0.021 (4)	0.592 (2)	0.068*	
H1WB	0.038 (2)	−0.002 (4)	0.5579 (17)	0.068*	
O2	−0.70407 (16)	0.6153 (2)	0.01860 (11)	0.0357 (5)	
O2W	0.16338 (18)	0.1231 (2)	0.40753 (11)	0.0324 (5)	
H2WA	0.149 (2)	0.061 (3)	0.3650 (11)	0.039*	
H2WB	0.207 (2)	0.071 (3)	0.4456 (11)	0.039*	
O3	−0.30374 (16)	0.1584 (2)	0.26291 (11)	0.0322 (4)	
O3W	0.13340 (17)	0.6755 (3)	0.41312 (11)	0.0331 (5)	
H3WA	0.183 (2)	0.732 (3)	0.4479 (12)	0.040*	
H3WB	0.120 (2)	0.730 (3)	0.3697 (10)	0.040*	
O4	−0.08994 (16)	0.4075 (2)	0.22554 (10)	0.0290 (4)	
O4W	0.12338 (18)	0.3724 (2)	0.52419 (11)	0.0306 (4)	
H4WA	0.092 (2)	0.452 (2)	0.5466 (16)	0.037*	
H4WB	0.103 (2)	0.276 (2)	0.5380 (16)	0.037*	
O5	−0.04711 (15)	0.3999 (2)	0.36330 (10)	0.0263 (4)	
O5W	0.33859 (16)	0.4349 (3)	0.45253 (12)	0.0348 (5)	
H5WA	0.400 (2)	0.396 (3)	0.4376 (16)	0.042*	
H5WB	0.357 (2)	0.520 (3)	0.4830 (15)	0.042*	
O6W	0.17072 (18)	0.3938 (2)	0.28435 (11)	0.0302 (4)	
H6WB	0.2178 (19)	0.467 (3)	0.2718 (16)	0.036*	
H6WA	0.1020 (16)	0.399 (3)	0.2526 (15)	0.036*	
N1	−0.34860 (18)	0.4432 (3)	0.22449 (12)	0.0223 (5)	
C1	−0.4197 (2)	0.5668 (3)	0.17514 (15)	0.0233 (6)	
H1A	−0.3972	0.6854	0.1826	0.028*	
C2	−0.5222 (2)	0.5226 (3)	0.11561 (14)	0.0224 (5)	
C3	−0.5541 (2)	0.3417 (3)	0.10731 (15)	0.0287 (6)	
H3A	−0.6260	0.3074	0.0687	0.034*	
C4	−0.4824 (2)	0.2166 (3)	0.15441 (15)	0.0279 (6)	
H4A	−0.5046	0.0981	0.1462	0.033*	
C5	−0.3744 (2)	0.2628 (3)	0.21590 (15)	0.0248 (6)	
C6	−0.2487 (2)	0.4933 (3)	0.29580 (14)	0.0269 (6)	
H6A	−0.2699	0.4489	0.3455	0.032*	
H6B	−0.2454	0.6214	0.2996	0.032*	
C7	−0.1195 (2)	0.4249 (3)	0.29292 (15)	0.0216 (5)	
C8	−0.6009 (2)	0.6622 (3)	0.06435 (14)	0.0240 (6)	

### Atomic displacement parameters (Å^2^)

**Table:**

	*U*^11^	*U*^22^	*U*^33^	*U*^12^	*U*^13^	*U*^23^
Zn1	0.02172 (18)	0.0210 (2)	0.02425 (17)	0.00083 (12)	0.00280 (12)	−0.00019 (12)
O1	0.0270 (10)	0.0199 (11)	0.0432 (11)	−0.0037 (8)	−0.0001 (8)	0.0073 (9)
O1W	0.0464 (15)	0.0551 (16)	0.0705 (17)	0.0124 (12)	0.0194 (12)	0.0136 (13)
O2	0.0303 (11)	0.0272 (11)	0.0393 (11)	−0.0024 (8)	−0.0125 (8)	−0.0002 (8)
O2W	0.0466 (13)	0.0209 (11)	0.0242 (10)	0.0051 (8)	−0.0032 (9)	−0.0027 (8)
O3	0.0332 (10)	0.0268 (11)	0.0346 (10)	0.0082 (8)	0.0040 (8)	0.0064 (8)
O3W	0.0361 (11)	0.0223 (11)	0.0341 (11)	0.0003 (8)	−0.0053 (9)	−0.0004 (8)
O4	0.0275 (10)	0.0371 (11)	0.0215 (9)	0.0010 (8)	0.0042 (8)	0.0006 (8)
O4W	0.0394 (12)	0.0296 (12)	0.0237 (10)	0.0038 (9)	0.0093 (8)	−0.0017 (8)
O5	0.0196 (9)	0.0366 (11)	0.0205 (9)	0.0013 (7)	0.0003 (7)	0.0007 (8)
O5W	0.0203 (10)	0.0372 (13)	0.0448 (12)	0.0009 (8)	0.0036 (9)	−0.0134 (9)
O6W	0.0278 (11)	0.0356 (12)	0.0258 (10)	−0.0059 (8)	0.0035 (8)	0.0019 (8)
N1	0.0183 (11)	0.0221 (13)	0.0242 (11)	0.0009 (9)	0.0002 (8)	−0.0009 (9)
C1	0.0237 (14)	0.0203 (15)	0.0260 (13)	0.0004 (10)	0.0058 (11)	0.0006 (11)
C2	0.0210 (13)	0.0208 (14)	0.0239 (13)	0.0004 (11)	0.0026 (10)	−0.0001 (11)
C3	0.0243 (14)	0.0286 (16)	0.0298 (14)	−0.0037 (11)	−0.0004 (11)	−0.0039 (12)
C4	0.0286 (14)	0.0187 (14)	0.0339 (14)	−0.0023 (11)	0.0025 (11)	−0.0032 (12)
C5	0.0255 (13)	0.0245 (16)	0.0262 (13)	0.0031 (11)	0.0096 (11)	0.0031 (11)
C6	0.0263 (14)	0.0267 (16)	0.0247 (13)	0.0027 (12)	−0.0002 (11)	−0.0034 (11)
C7	0.0221 (13)	0.0142 (14)	0.0267 (13)	−0.0036 (10)	0.0021 (10)	−0.0005 (10)
C8	0.0225 (13)	0.0264 (16)	0.0223 (13)	0.0027 (11)	0.0034 (10)	0.0002 (11)

### Geometric parameters (Å, °)

**Table:**

Zn1—O5W	2.0554 (17)	O5W—H5WA	0.821 (16)
Zn1—O6W	2.0759 (18)	O5W—H5WB	0.812 (16)
Zn1—O4W	2.0805 (18)	O6W—H6WB	0.816 (15)
Zn1—O2W	2.0945 (18)	O6W—H6WA	0.814 (16)
Zn1—O3W	2.0988 (19)	N1—C1	1.362 (3)
Zn1—O5	2.1039 (16)	N1—C5	1.392 (3)
O1—C8	1.251 (3)	N1—C6	1.464 (3)
O1W—H1WA	0.829 (17)	C1—C2	1.355 (3)
O1W—H1WB	0.812 (17)	C1—H1A	0.9300
O2—C8	1.257 (3)	C2—C3	1.409 (4)
O2W—H2WA	0.832 (15)	C2—C8	1.498 (3)
O2W—H2WB	0.802 (15)	C3—C4	1.356 (3)
O3—C5	1.246 (3)	C3—H3A	0.9300
O3W—H3WA	0.813 (15)	C4—C5	1.416 (3)
O3W—H3WB	0.816 (15)	C4—H4A	0.9300
O4—C7	1.245 (3)	C6—C7	1.518 (3)
O4W—H4WA	0.823 (15)	C6—H6A	0.9700
O4W—H4WB	0.809 (15)	C6—H6B	0.9700
O5—C7	1.267 (3)		
			
O5W—Zn1—O6W	92.57 (8)	H6WB—O6W—H6WA	110 (2)
O5W—Zn1—O4W	89.79 (8)	C1—N1—C5	122.35 (19)
O6W—Zn1—O4W	172.96 (8)	C1—N1—C6	121.7 (2)
O5W—Zn1—O2W	93.41 (8)	C5—N1—C6	115.59 (19)
O6W—Zn1—O2W	88.53 (7)	C2—C1—N1	122.1 (2)
O4W—Zn1—O2W	84.70 (7)	C2—C1—H1A	118.9
O5W—Zn1—O3W	86.50 (7)	N1—C1—H1A	118.9
O6W—Zn1—O3W	96.52 (7)	C1—C2—C3	117.1 (2)
O4W—Zn1—O3W	90.25 (7)	C1—C2—C8	120.8 (2)
O2W—Zn1—O3W	174.95 (8)	C3—C2—C8	122.0 (2)
O5W—Zn1—O5	171.74 (7)	C4—C3—C2	121.5 (2)
O6W—Zn1—O5	91.03 (7)	C4—C3—H3A	119.3
O4W—Zn1—O5	87.51 (7)	C2—C3—H3A	119.3
O2W—Zn1—O5	94.10 (7)	C3—C4—C5	121.4 (2)
O3W—Zn1—O5	85.71 (6)	C3—C4—H4A	119.3
H1WA—O1W—H1WB	107 (3)	C5—C4—H4A	119.3
Zn1—O2W—H2WA	123.1 (17)	O3—C5—N1	118.3 (2)
Zn1—O2W—H2WB	122.1 (18)	O3—C5—C4	126.2 (2)
H2WA—O2W—H2WB	111 (2)	N1—C5—C4	115.5 (2)
Zn1—O3W—H3WA	120.8 (18)	N1—C6—C7	114.4 (2)
Zn1—O3W—H3WB	116.6 (19)	N1—C6—H6A	108.7
H3WA—O3W—H3WB	109 (2)	C7—C6—H6A	108.7
Zn1—O4W—H4WA	121.7 (18)	N1—C6—H6B	108.7
Zn1—O4W—H4WB	118.0 (19)	C7—C6—H6B	108.7
H4WA—O4W—H4WB	111 (2)	H6A—C6—H6B	107.6
C7—O5—Zn1	132.66 (16)	O4—C7—O5	125.4 (2)
Zn1—O5W—H5WA	131.2 (19)	O4—C7—C6	120.3 (2)
Zn1—O5W—H5WB	114.9 (18)	O5—C7—C6	114.1 (2)
H5WA—O5W—H5WB	112 (2)	O1—C8—O2	124.0 (2)
Zn1—O6W—H6WB	117.0 (19)	O1—C8—C2	118.4 (2)
Zn1—O6W—H6WA	109.4 (19)	O2—C8—C2	117.6 (2)
			
O5W—Zn1—O5—C7	103.1 (5)	C1—N1—C5—C4	−2.3 (3)
O6W—Zn1—O5—C7	−12.8 (2)	C6—N1—C5—C4	171.2 (2)
O4W—Zn1—O5—C7	174.1 (2)	C3—C4—C5—O3	178.9 (2)
O2W—Zn1—O5—C7	−101.4 (2)	C3—C4—C5—N1	0.5 (4)
O3W—Zn1—O5—C7	83.6 (2)	C1—N1—C6—C7	−121.4 (2)
C5—N1—C1—C2	1.6 (4)	C5—N1—C6—C7	65.1 (3)
C6—N1—C1—C2	−171.5 (2)	Zn1—O5—C7—O4	21.6 (4)
N1—C1—C2—C3	1.0 (3)	Zn1—O5—C7—C6	−154.06 (16)
N1—C1—C2—C8	177.9 (2)	N1—C6—C7—O4	32.4 (3)
C1—C2—C3—C4	−2.8 (4)	N1—C6—C7—O5	−151.6 (2)
C8—C2—C3—C4	−179.6 (2)	C1—C2—C8—O1	9.1 (3)
C2—C3—C4—C5	2.1 (4)	C3—C2—C8—O1	−174.2 (2)
C1—N1—C5—O3	179.1 (2)	C1—C2—C8—O2	−170.0 (2)
C6—N1—C5—O3	−7.4 (3)	C3—C2—C8—O2	6.7 (3)

### Hydrogen-bond geometry (Å, °)

**Table:**

*D*—H···*A*	*D*—H	H···*A*	*D*···*A*	*D*—H···*A*
O1W—H1WA···O2^i^	0.83 (2)	2.25 (2)	2.972 (3)	145 (3)
O1W—H1WB···O2W^ii^	0.81 (2)	2.58 (3)	3.118 (3)	125 (3)
O2W—H2WA···O4^iii^	0.83 (2)	1.89 (2)	2.716 (2)	169 (3)
O2W—H2WB···O2^i^	0.80 (2)	1.96 (2)	2.742 (2)	163 (2)
O3W—H3WA···O2^iv^	0.81 (2)	1.89 (2)	2.701 (2)	173 (2)
O3W—H3WB···O4^v^	0.82 (2)	2.04 (2)	2.849 (2)	170 (3)
O4W—H4WA···O5^vi^	0.82 (2)	2.02 (2)	2.810 (2)	160 (3)
O5W—H5WA···O1^iii^	0.82 (2)	1.90 (2)	2.705 (2)	166 (3)
O5W—H5WB···O1^iv^	0.81 (2)	1.95 (2)	2.742 (2)	164 (3)
O6W—H6WB···O3^v^	0.82 (2)	1.89 (2)	2.697 (3)	171 (3)

Symmetry codes: (i) *x*+1, −*y*+1/2, *z*+1/2; (ii) −*x*, −*y*, −*z*+1; (iii) −*x*, *y*−1/2, −*z*+1/2; (iv) *x*+1, −*y*+3/2, *z*+1/2; (v) −*x*, *y*+1/2, −*z*+1/2; (vi) −*x*, −*y*+1, −*z*+1.
